# SU^2^GE-Net: a saliency-based approach for non-specific class foreground segmentation

**DOI:** 10.1038/s41598-023-40175-9

**Published:** 2023-08-15

**Authors:** Xiaochun Lei, Xiang Cai, Linjun Lu, Zihang Cui, Zetao Jiang

**Affiliations:** 1 School of Computer Science and Information Security, Guilin University of Electronic Technology, GuiLin, 541010 Guangxi China; 2 Guangxi Key Laboratory of Image and Graphic Intelligent Processing, Guilin University of Electronic Technology, Guilin, 541004 Guangxi China

**Keywords:** Engineering, Mathematics and computing

## Abstract

Salient object detection is vital for non-specific class subject segmentation in computer vision applications. However, accurately segmenting foreground subjects with complex backgrounds and intricate boundaries remains a challenge for existing methods. To address these limitations, our study proposes SU^2^GE-Net, which introduces several novel improvements. We replace the traditional CNN-based backbone with the transformer-based Swin-TransformerV2, known for its effectiveness in capturing long-range dependencies and rich contextual information. To tackle under and over-attention phenomena, we introduce Gated Channel Transformation (GCT). Furthermore, we adopted an edge-based loss (Edge Loss) for network training to capture spatial-wise structural details. Additionally, we propose Training-only Augmentation Loss (TTA Loss) to enhance spatial stability using augmented data. Our method is evaluated using six common datasets, achieving an impressive $F_{\beta }$ score of 0.883 on DUTS-TE. Compared with other models, SU^2^GE-Net demonstrates excellent performance in various segmentation scenarios.

## Introduction

Deep learning has been applied to all sectors^1,2^ in recent years. Image segmentation is a new task based on deep learning techniques. Image segmentation^3–6^ is widely used in various fields, such as autonomous driving and portrait photography. The core segmentation methods are all based on datasets limited to single or multiple categories for segmentation, and the semantic segmentation task can be combined with the saliency object detection task to achieve a non-specific class of foreground segmentation. The pixel-level semantic segmentation task is transformed into a binary classification problem that distinguishes whether the pixel points of an image belong to the foreground or the background. Such algorithms can be applied to intelligent media interaction to quickly design creative images that can change backgrounds for pictures or videos and integrate foreground characters into different scenes to produce various creative applications.

**Figure 1:**
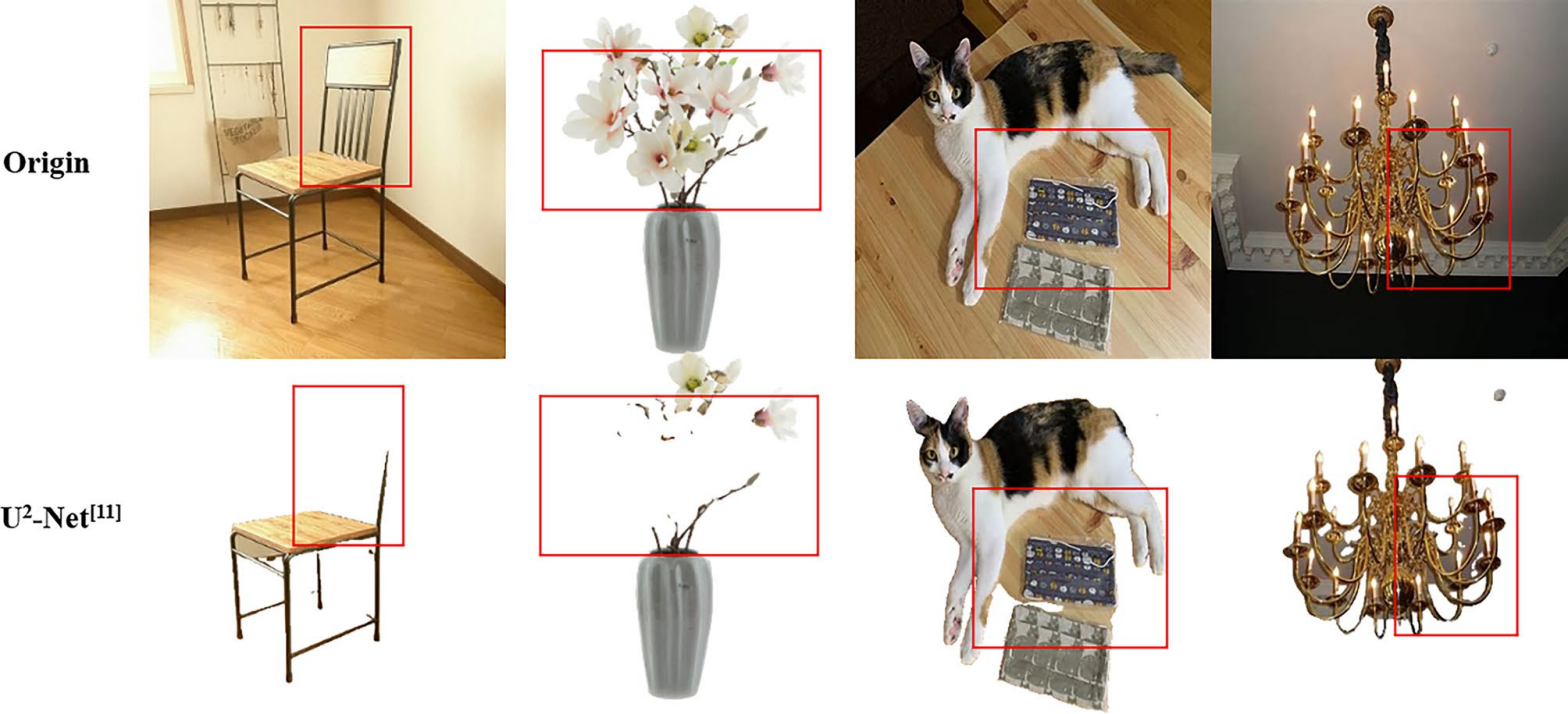
Segmentation results of U^2^-Net.

U^2^-Net^7^ is currently one of the state-of-the-art methods in the field of saliency object detection, but using a direct method is not feasible in non-specific class subject segmentation. Some of the results are demonstrated in the central column of Fig. 1, the problems of which include:

1. The subject information will be lost when the foreground subject is not continuous.
2. It is difficult to separate the background and foreground subjects for skeleton objects or objects close to the background color.
3. The problem of missing subjects occurs in the case of multiple subjects.

U^2^-Net does not focus enough attention on the subject leading to the first problem, which can be resolved using the GCT^8^. The GRSU-L module is constructed by integrating the GCT with the RSU-L module of U^2^-Net. This module uses learnable parameters to decide the channel relationship of the feature map. These weight parameters determine the competition and cooperative behavior between neurons and are jointly optimized with the convolution weight. This improved scheme can detect the main part more acutely.

The simplified work requires only the foreground and background of the dichotomous image. Edge extraction is relatively difficult for complex RGB images but relatively easy for edges of binary images, so Edge Loss is proposed to resolve the second problem. Edge Loss performs edge detection on the segmented mask and label mask and calculates the binary cross-entropy loss to obtain the difference between the predicted result and the label on the edge, enabling the model to learn along the direction of the accurate edge.

The first GRSU-L module severely affects the subsequent detection task by extensive experiments. When there is a large missing area in the detection result, the feature map of the first GRSU-L module is already missing that part and is not caused by downsampling. Therefore, the third problem should be solved by enhancing the spatial perceptibility and global context-aware of the model. TTA Loss is proposed to solve this problem. By fusing the prediction after data enhancement (for example, horizontal flipping) using the spatial domain with the prediction of the original images and back-propagating the loss calculated as the final prediction result, the model converges faster and better. The Transformer is better than CNN in obtaining global features, so the Swin TransformerV2 is added to obtain more global information.

The final modified network SU^2^GE-Net achieves the foreground segmentation of non-specific class of image subjects. In addition, experimental validation is performed on DUTS-TE^9^, ECSSD^10^, PASCAL-S^11^, HKU-IS^12^, DUT-OMRON^13^, SOD^14^, showing that the proposed model outperforms well.

The main contributions of the paper are:

1. A saliency-based foreground subject segmentation model, SU^2^GE-Net, is proposed to effectively separate non-specific class foregrounds from backgrounds.
2. Based on U^2^-Net, the Swin TransformerV2^15^ is used for feature extraction. SU^2^GE-Net is rebuilt according to the architecture of U^2^-Net, which improves the performance of extracting image features from the backbone network. The GRSU-L module was reconstructed by integrating the GCT to achieve a better segmentation effect non-specific categories of subjects.
3. TTA Loss and Edge Loss are added to the model training process to improve the model convergence efficiency, optimize the edge detail part and subject recognition.

## Related works

### Salient object detection

Salient object detection is similar in results to that of the segmentation task, with the difference in the different Ground Truth labels of the two tasks. The purpose of salient object detection is to identify the main part and analyze the probability that each pixel in the image is the main class. The real label of the segmentation task marks the category corresponding to each pixel.

Salient object detection can be divided into two categories according to different data types, one is salient object detection of RGB image type, whose input image is the common RGB image type, and the other is salient object detection of RGB-D^16–18^ image type, whose input image includes depth maps in addition to RGB images. Since depth maps require an additional depth camera to capture, only RGB images are considered for salient object detection to acquire the main part of the image.

U^2^-Net uses an Encoder–Decoder structure for salient object detection of images. The U^2^-Net encoder has been strategically designed to make feature extraction more efficient and rich to better distinguish the main part.

### Non-specific class foreground subject segmentation

The task of non-specific class foreground subject segmentation is to find and segment the main part of an input image. This task is also very similar to the matting task^19^, which starts by feeding the trimap together to the Encoder–Decoder, predicting the alpha mask of the image, and optimizing the alpha mask with a small network for more detailed edges.

Sengupta et al.^20^ have proposed a background matting technique that enables casual capture of high-quality foreground+alpha mattes in natural settings. This approach avoids using a green screen or painstakingly constructing a detailed trimap as typically needed for high matting quality.

However, the only disadvantage is that the model needs the background image of the input image. The background image is not easy to obtain, so the scheme also has some limitations. Chen et al.^21^ achieved excellent results in portrait segmentation with a single RGB image input, but unfortunately, the method only targets a single category and does not achieve foreground subject segmentation for a non-specific class.

### Attention mechanism

Attention mechanisms in computer vision are implemented in various forms, such as channel attention mechanisms^22–24^, spatial attention mechanisms^25,26^, self-attention mechanisms^27,28^, and gated attention mechanisms^10,29^. Channel attention and spatial attention, respectively, set different weights that can be learned at the channel and spatial levels of an image and use these weights to distinguish the importance of different channels and spaces. The self-attention mechanism disregards pooling weights and instead employs mappings of feature maps to distinct spaces, combining features from three different spaces in a specific manner to achieve the attention mechanism’s intended effect. Gated attention, however, uses learnable parameters to model the channel relations in the feature map, which correspond to the competition and cooperation relations of neurons in the neural network, and guides the competition and cooperation by gating parameters, thus solving the deficiency of insufficient attentional attention.

### Swin TransformerV2

Compared to CNN, the Transformer^30^ can extract global features better. The Swin TransformerV2, modified from the Swin Transformer^31^, makes the network model larger and can adapt to different resolution images and different size windows.

### Test time augmentation

Test Time Augmentation^32^ is a trick that is recognized to improve predictions and is often used in hit-list competitions. Specifically, it creates multiple augmented copy images of each predicted image in the test set, lets the model make predictions for each image, and speaks the corresponding images for fusion as the final prediction. Although Test Time Augmentation can get better prediction results, it increases the time consumed, so we propose TTA Loss. The process is described in the section Training-only Augmentation Loss.

## Proposed method

**Figure 2:**
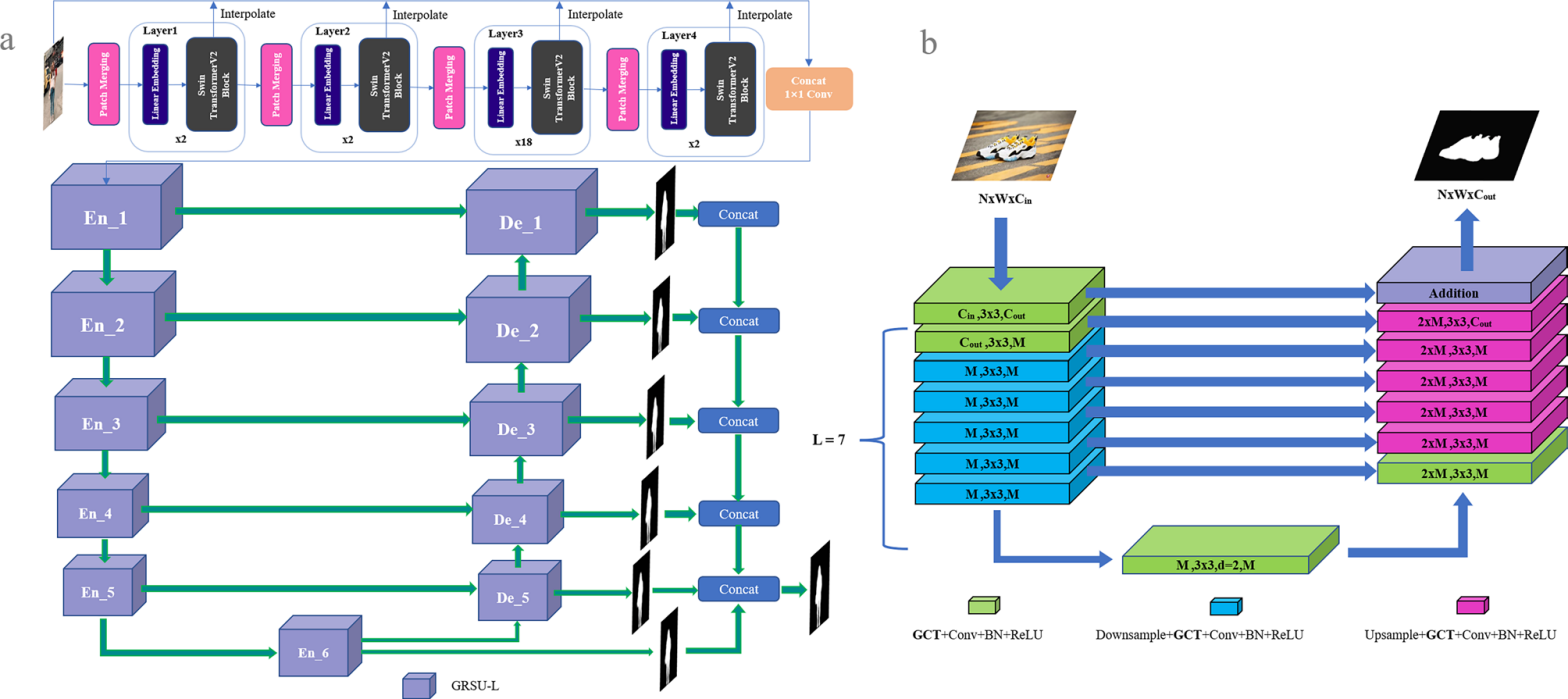
Network structure chart: (**a**) the SU^2^GE-Net structure and (**b**) the GRSU-L module structure.

Initially, we present an overview of the utilized modules and elaborate on the specifics of the SU^2^GE-Net network architecture in Fig. 2a. The network supervision strategy and the loss are described at the end of this section.

### Swin TransformerV2

The Swin TransformerV2 tackles three major issues in the training and application of large vision models. A residual-post-norm method combined with cosine attention was used to improve training stability uses. It proposes a log-spaced continuous position bias method to effectively transfer models pre-trained using low-resolution images to downstream tasks with high-resolution inputs. The Transformer requires a large dataset and has a large computational; therefore, the Swin TransformerV2 uses a self-supervised pre-training method, SimMIM, to reduce the need for vast labeled images.We tested various Swin TransformerV2 models on DUTS-TE and ended up using the following config: input size:256, drop path rate:0.3, embed dim:96, depths:[2, 2, 18, 2], num heads:[3, 6, 12, 24], window size:16.

### GRSU-L module

The GRSU-L module is the basic unit that constitutes the SU^2^GE-Net, and its internal RSU-L structure is the same as that of U^2^-Net, a U-shaped structure. The structure diagram of the GRSU-L module is illustrated in Fig. 2b. GCT denotes the module that implements the GCT. To solve the attention problem, the GCT is introduced in the GRSU-L module to obtain the most attentional part of the image before performing feature extraction. It is set before each convolutional layer of GRSU-L so that the module can extract more attentional features^33^. The regions with higher attention weights are often the main part of the image, and the use of gated attention enables the model to segment the main part better. In the GRSU-L module, L denotes the number of layers that the module performs in the Encoder–Decoder phase. By controlling the number of layers and the extraction method used for feature extraction of feature maps in different stages of the network, effective utilization of features for images of different scales is achieved.

### Gated channel transformation (GCT)

**Figure 3:**
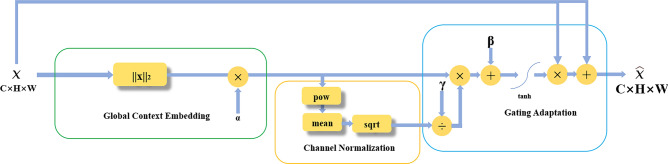
The GCT structure.

The utilization of GCT addresses the model attention problem by leveraging the competitive and cooperative dynamics among neurons in neural networks. This mechanism stimulates collaboration among network neurons when attention is insufficient, enhancing focus on the subject of interest. Conversely, it encourages competition among neurons in situations of excessive attention, facilitating the retention of more competitive components. The GCT is illustrated in Fig. 3.

### Architecture of SU^2^GE-Net

In order to enhance our model’s ability to capture long-range dependencies and extract comprehensive contextual information, we have opted to substitute the conventional CNN-based backbone with the Swin-TransformerV2. Additionally, GCT was introduced to autonomously regulate the interplay between competition and cooperation among neurons during the model training process. This guidance facilitated the model in prioritizing its attention toward the main component. The GRSU-L module constructed by the GCT, as the basic unit of the SU^2^GE-Net, has an internal U-shaped Encoder–Decoder structure. The extracted features are different according to the different depths of the network. The SU^2^GE-Net is a two-level nested U-structure, and combined with the reasonable use of the GRSU-L module; it can obtain different scales of the segmentation results (6 scales are used in the article) extracted again by 1 $\times$ 1 convolution after stitching the 6 different scales of feature maps. To address the problem of rough object edge segmentation, edge detection is performed using pairs of segmentation results with the results of real labels, and the difference between the two edges is calculated using Binary Cross-Entropy loss. The gradient calculation is performed using TTA Loss, which eventually guides the network to output a more refined network segmentation of the subject. The introduction of this loss function will only guide the segmentation results of the image subject segmentation model toward fine edges during the training process of the model and will not increase the number of parameters or the computational effort of the model. We sample the output of each Swin TransformerV2 Block back to the size of the original images, concatenate with the original images, and later use 1$\times$1 convolution to downscale to three channels. Figure 2a shows the SU^2^GE-Net structure diagram in detail.

### Edge loss

Following the loss function design adopted by U^2^-Net, the outer U-shaped structure, the subject mask generated by each layer of decoding, and the real labels are computed with a Binary Cross-Entropy loss for deep supervised training, and this loss $L_{global}$ can be expressed in Eq. (1):

1 $$\begin{aligned} L_{\text {global }}=\sum _{i=1}^N {\text {BCELoss}}\left( x_i, y_i\right) , \end{aligned}$$

where *N* denotes the total number of layers in the outer U-shaped structure, and *i* denotes the subject mask output at layer *i* in the decoder. $x_i$ represents the predicted result. Respectively, $y_i$ denotes the true label corresponding to the input image.

In the model calculation process, the size of the output result of each layer is different, so the images are interpolated to the size of the input image before summation is performed. *BCELoss*() denotes the Binary Cross-Entropy loss function, which is calculated as depicted in Eqs. (2) and (3):

2 $$\begin{aligned} B C E L o s s(a, b)= & \, {\text {mean}}\left( \left\{ l_1, \ldots , l_S\right\} ^{\top }\right) , \end{aligned}$$

3 $$\begin{aligned} l_j= & {} -w_j\left[ b_j \cdot \log a_j+\left( 1-b_j\right) \cdot \log \left( 1-a_j\right) \right] . \end{aligned}$$

*mean*() denotes the average of all *l*. Where $a_j$ and $b_j$ denote the pixel value of the jth input image with its corresponding label, BatchSize is *S*, and $w_j$ denotes the weight of the jth image.

To address the problem that the segmentation of object edges by U^2^-Net has certain defects, the edge operator *Edge()* is used to perform edge detection of the prediction mask and the real label, and the Edge Loss is designed according to the edge detection results of both, whose calculation can be expressed using Equation (4):

4 $$\begin{aligned} L_{\text {edge }}\left( x_0, y_0\right) =BCE {\text {Loss}}\left( {\text {Edge}}\left( x_0\right) , {\text {Edge}}\left( y_0\right) \right) , \end{aligned}$$

where $x_0$ and $y_0$ denote the mask and true label of the final subject segmentation of the model, respectively, and *Edge*() denotes the edge detection of the input image using edge operators (e.g. Canny, Laplacian, Sobel, Scharr).

The loss function Loss for model training is obtained by combining $L_{edge}$ and $L_{global}$ as shown in Eq. (5):

5 $$\begin{aligned} \text { Loss }=w_g \cdot L_{\text {global }}+w_e \cdot L_{\text{ edge }}. \end{aligned}$$

**Figure 4:**
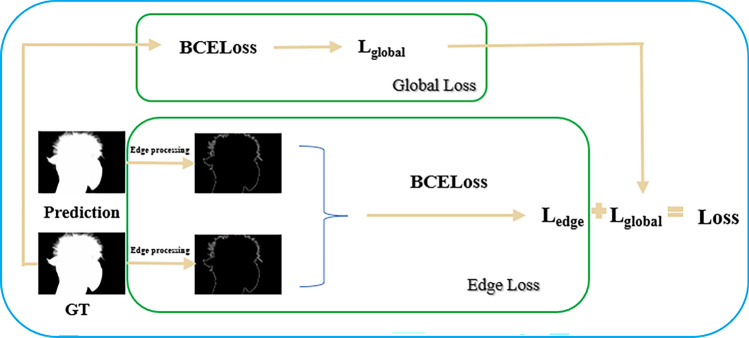
The pipeline of calculating edge loss.

Among them, $w_g$ and $w_e$ are hyperparameters that can be set independently. In the early training period, the foreground segmentation model of non-specific class subjects is not perfect, and the edge operator extracts the image edges poorly, so the value of $w_g$ will be set larger than $w_e$, whereas the segmentation results are gradually refined in the later training period, so the value of $w_g$ will be set smaller than $w_e$. The process is illustrated in Fig. 4.

### Training-only augmentation loss

**Figure 5:**
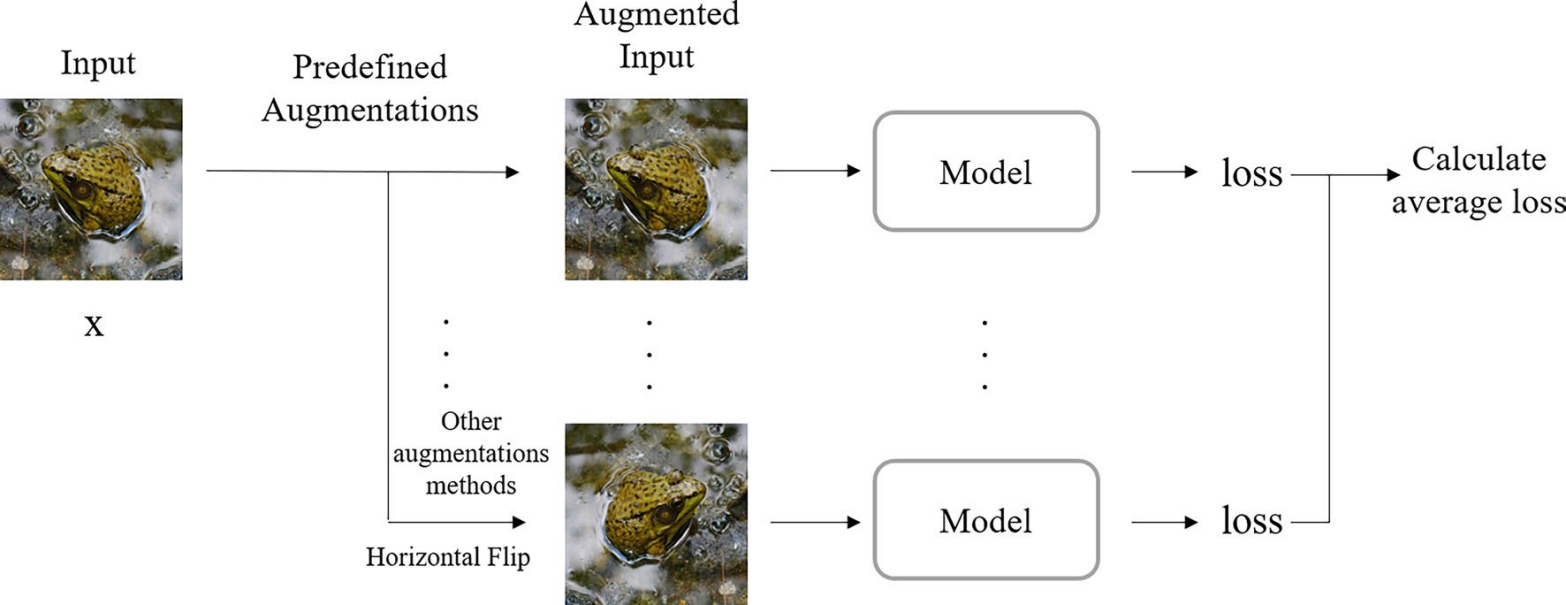
The TTA loss calculation pipeline.

In a manner similar to Test Time Augmentation, input *x* performs data augmentation again before inputting into the network, the results of multiple data augmentations are predicted and the corresponding losses are obtained, and the mean of these losses is used to back-propagation to provide a more accurate convergence guide to the model. The procedure is depicted in Fig. 5. $Loss_{tta}$ is referred to as Eq. (6):

6 $$\begin{aligned} {\text {Loss}}_{t t a}=A v e\left( {\text {Loss}}\left( \sum _{x \in X} n e t(x)\right) , g t\right) , \end{aligned}$$

where *net*() is SU^2^GE-Net, *gt* is the Ground Truth, *x* is the set of predicted images containing the original images and the data enhancement of the original images, the data enhancement can be flipped, rotated shifted, etc. *Ave*() is the averaging function. We believe that the idea of TTA Loss can be used in various tasks and is not limited to saliency target detection. $Loss_{tta}$ is the value ultimately used in the article to guide SU^2^GE-Net back-propagation.

## Results and discussion

### Dataset

Train set: We use DUTS-TR to train SU^2^GE-Net. DUTS-TR contains 10,553 images and is generally used on saliency object detection. Test set: SOD includes 300 images that were originally intended for image segmentation. ECSSD contains 1000 images that are semantically meaningful but structurally complex. DUT-OMRON has 5168 images, each with one or two objects. PASCAL-S is made up of 850 images with cluttered backgrounds and intricate foreground objects. HKU-IS has 4447 images. The majority of them have multiple connected or disconnected foreground objects. DUTS-TE is a part of DUTS and has 5019 images for testing.

### Implementation details

Training was performed on a Tesla A100 GPU (40GB), where the images were first scaled to a size of 320 $\times$ 320, then horizontally flipped for data enhancement, and finally randomly cropped to a size of 288 $\times$ 288. The hyperparameters $w_g$ and $w_e$ in the total loss function are set to 0.7 and 0.3 in the first 100 epochs and swapped with each other in the second 100 epochs. Using an AdamW optimizer with OneCycleLR as Schedule, the maximum learning rate was set to 1e−5, betas = (0.9, 0.999).eps = 1e−8, $weight_{decay}$ = 0.05. The results of the metrics calculation are illustrated in Table 1, and the metrics were calculated once per 1000 iterations. TTA Loss was used after training 20 epochs.

### Evaluation metrics

In our evaluation, we employed five widely adopted metrics, namely *MAE*, *MaxF*, *MeanF*, $F_\beta$ and $S{\text{-}}measure$, to assess the performance of the model. *MaxF*, *MeanF*, and $F_\beta$ were computed based on precision-recall pairs, using a weight $\beta ^2$ of 0.3. MaxF represents the maximum value achieved across all thresholds, while *MeanF* denotes the average value calculated for all thresholds. For this particular case, $F_\beta$ was determined using the middle threshold of 127. The $S{\text{-}}measure$ incorporates two components: object-aware (So) and region-aware (Sr), both weighted equally with $\alpha$ set to 0.5 to ensure equilibrium.

### Tests with different edge operators

**Figure 6:**
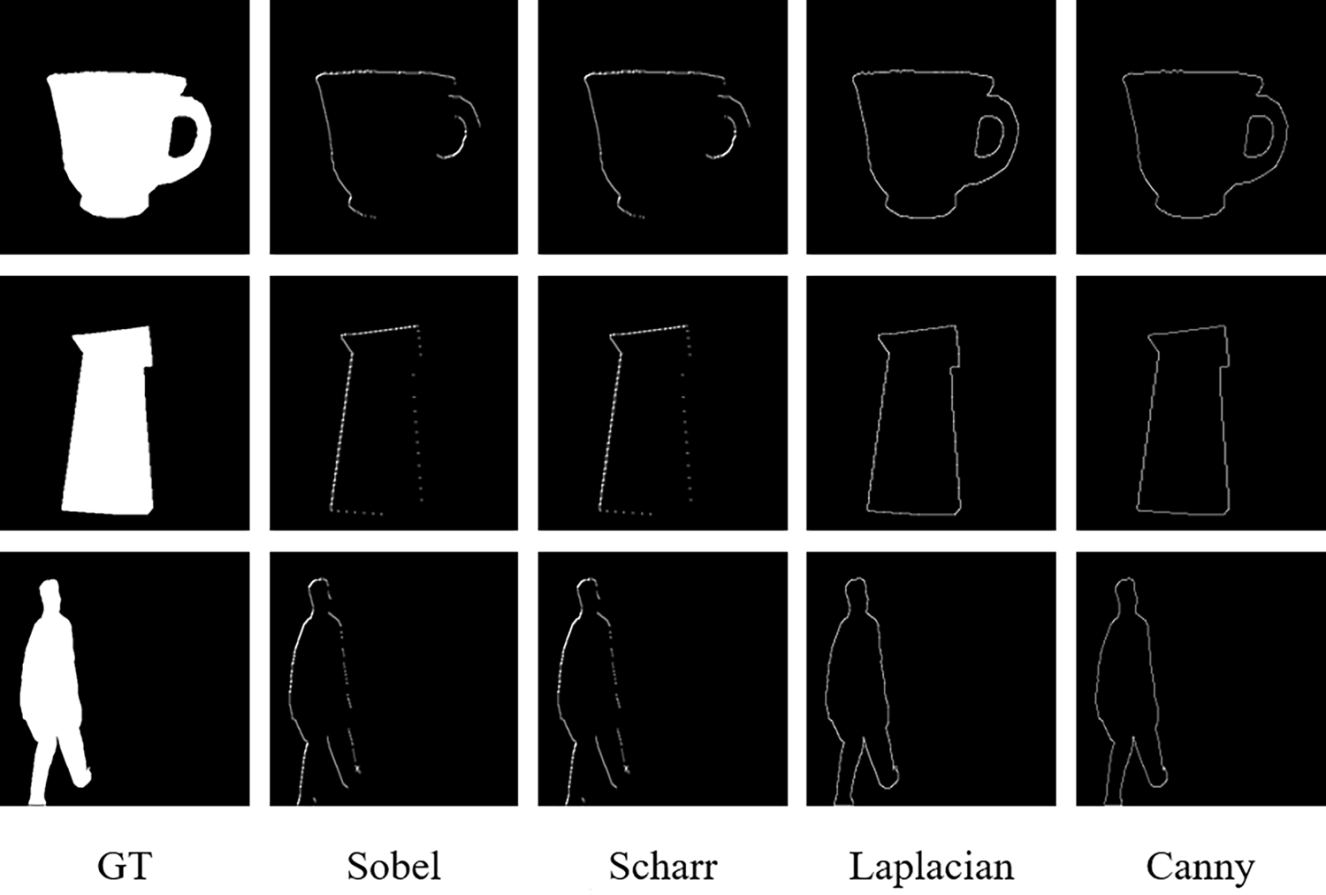
Detection results of different edge detection operators.

In this subsection, four operators, Sobel, Scharr, Laplacian, and Canny, were used for the Edge Loss function of SU^2^GE-Net in the Edge Loss calculation, and the model was trained separately to determine the most suitable operator in the Edge Loss function. After the input image was downsampled by the model, the resolution decreased, making the edges of the image not clear enough; even if the image is restored using the upsampling method, the original edges of the image also have some loss. Since the real label is not downsampled, the edge information is not lost. The article designs an Edge Loss function by performing edge detection on the output mask and the real label (both are binarized images) and using the edge information not lost in the real label as the basis for the edge refinement of the output mask. The traditional edge detection operator, which usually has a better performance in images without complex pixels, especially in binarized images, and the edge detection results of some binarized images are displayed in Fig. 6.

Sobel and Scharr are first-order operators, while Laplacian and Canny are improved second-order operators built on top of the first-order. The second-order operators usually process better than the first-order operators. In DUTS-TE there are more subjects with complex structures which have more noise on the edges, and Canny is generally sensitive to noise, and the results of image processing for most of them are not very different and more stable, and Canny has also achieved better results in the experiments. Therefore, in the training of SU^2^GE-Net, the Canny operator was used to calculate Edge Loss.

### Comparison with state-of-the-arts

We compare the proposed algorithm with 6 state-of-the-art saliency detection methods, including the U^2^-Net, BASNet^34^, P2T^35^, MSIN^36^, SCRN^37^, EGNet-R^38^,RCSB^39^ and DC-Net^40^. All saliency maps of these methods are computed by their released codes for fair comparisons. The superiority of SU^2^GE-Net can be seen in Table 1.

### Qualitative evaluation

**Figure 7:**
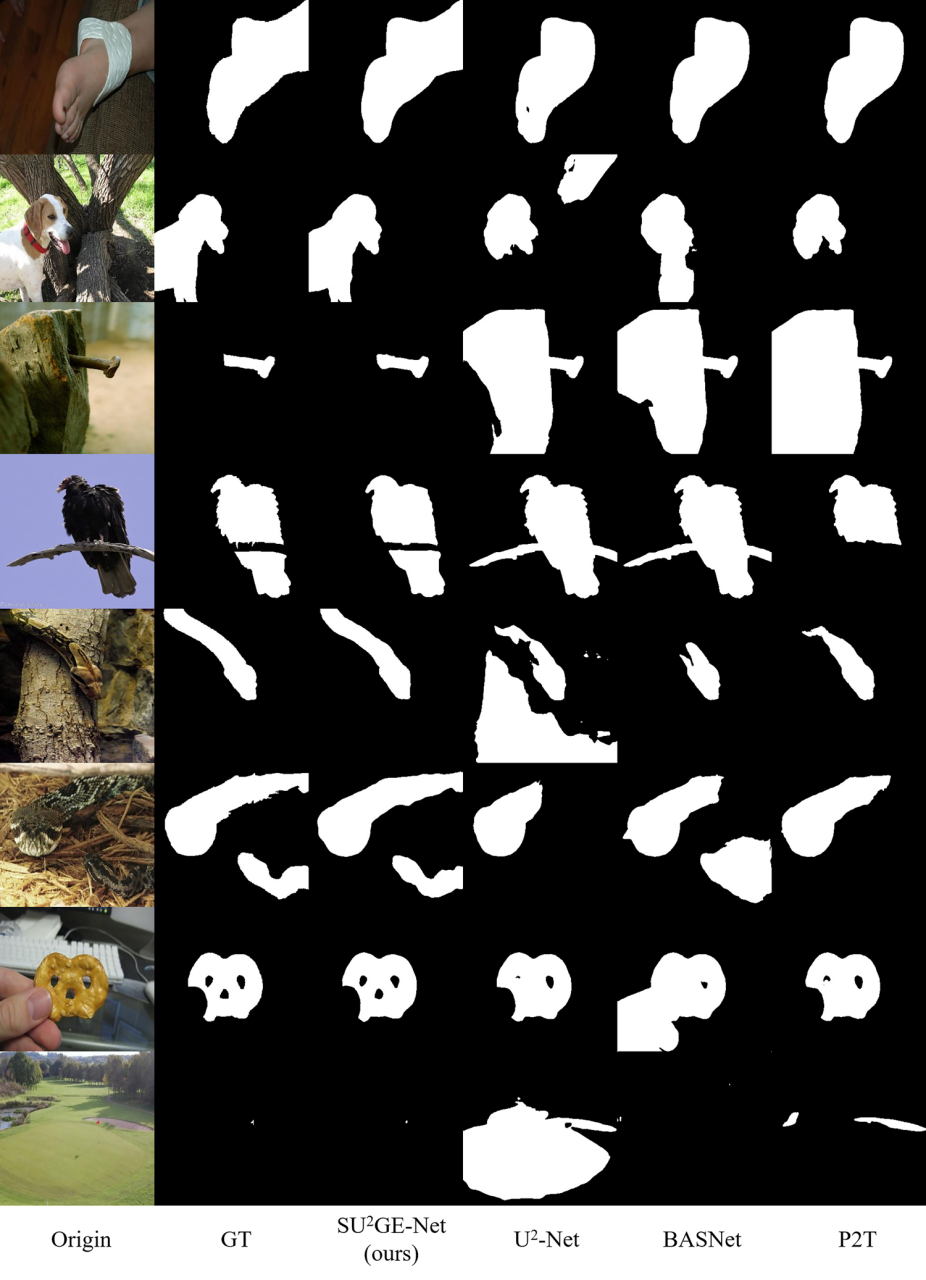
Several visual examples with style-varying objects and their predictions generated by the proposed SU^2^GE-Net, U^2^-Net, U^2^-Net, BASNet and P2T methods.

Some representative examples are shown in Fig. 7. These examples reflect a variety of situations. 1st to 3rd row reflects the recognition and segmentation of the subject in different situations. Compared to the 1st row, all other models result in missing segmentation above the ankle part due to the white harness. And SU^2^GE-Net segments the edges more smoothly than *gt*. For the 2nd row, other models fail to distinguish the subject due to changes in the color of the dog’s fur and the overlapping of the tree and dog, resulting in incorrect segmentation. SU^2^GE-Net completely distinguishes the body part of the dog. For the prediction of the 3rd row, other models inaccurately segmented the subject due to the similar color of the rusty nail and the tree stump. SU^2^GE-Net is able to extract the main body of the iron nail. 4th row reflects the segmentation effect of the object on the occluded subject. U^2^-Net and BASNet incorrectly identify the branch as the subject, while P2T predicts the branch as the background but does not recognize the tail well due to the lack of global semantic information. SU^2^GE-Net is able to remove the branches and retain the bird as a whole. The 5th and 6th rows show the segmentation of single and multiple objects with low contrast between foreground and background. Even when the subject is challenging to identify with eyes, SU^2^GE-Net can still be effectively segmented. The remaining models can only segment a single object, and some of the segmentation is erroneous. In assumption, SU^2^GE-Net consistently generates more accurate and complete saliency maps, can effectively segment holes and occluded objects, and identifies borders with low contrast and small objects.

### Ablation experiment

**Figure 8:**
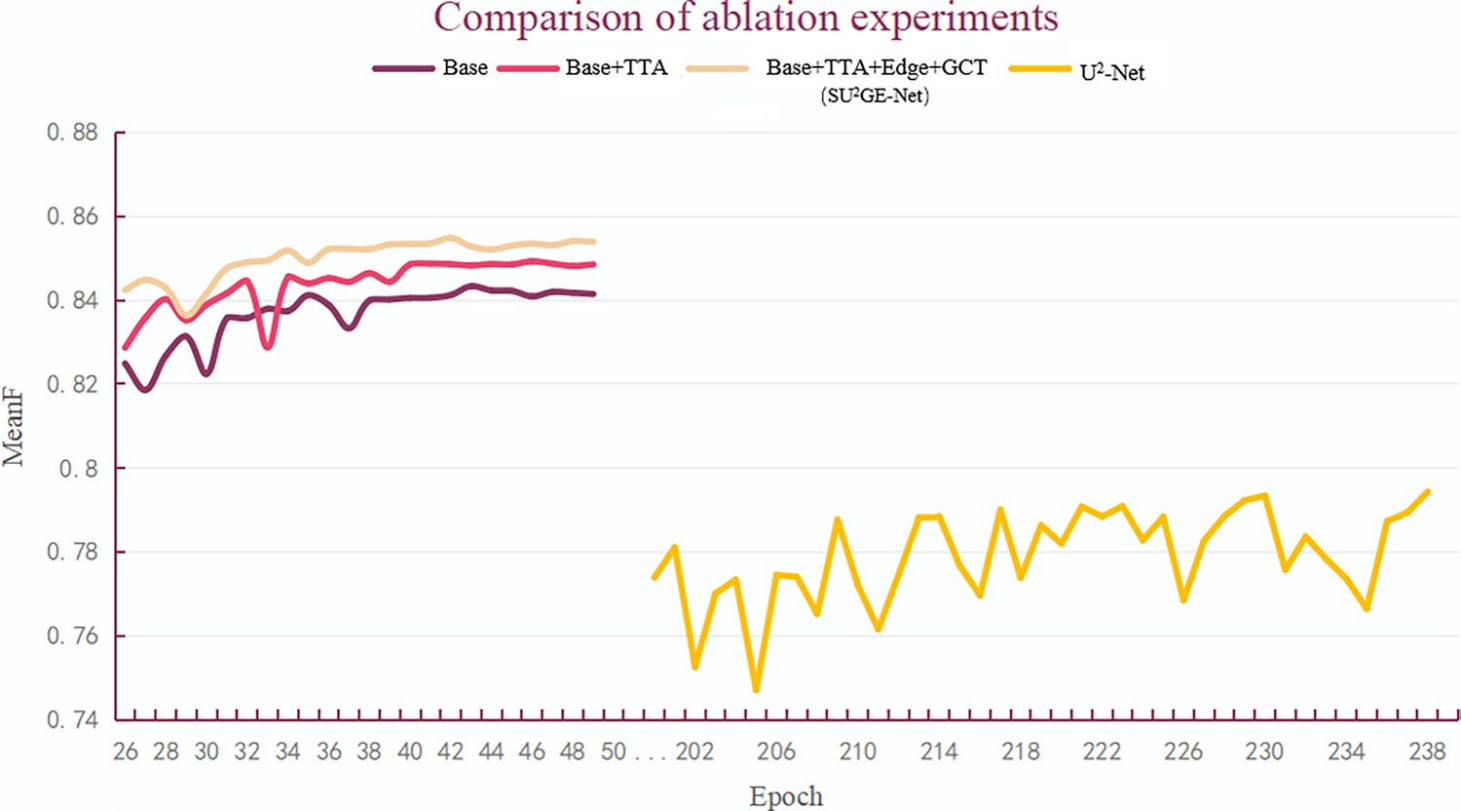
Training process MeanF curves.

The effectiveness of the different trick overlays can be seen in Table 1. Figure 8 shows the ablation experiments in three different groups. The comparison of the proposed methods and U^2^-Net demonstrates that our methods perform admirably in terms of convergence speed. The Base is U^2^-Net+Swin TransformerV2, and the other two groups build on it by adding TTA loss, Edge Loss, and GCT. It is obvious to see that the model is almost converged in the 45th epoch, while U^2^-Net needs about 230 epochs.

**Table 1:** Metrics on the DUTS-TE, DUT-OMRON, HKU-IS, ECSSD, SOD, and PASCAL-S test sets were calculated.

Model				DUTS-TE					DUT-OMRON					HKU-IS				
Swin	TTA	Edge	GCT	MAE$\downarrow$	MaxF$\uparrow$	MeanF$\uparrow$	$F_{\beta }\uparrow$	S-measure$\uparrow$	MAE$\downarrow$	MaxF$\uparrow$	MeanF$\uparrow$	$F_{\beta }$ $\uparrow$	S-measure$\uparrow$	MAE$\downarrow$	MaxF$\uparrow$	MeanF$\uparrow$	$F_{\beta }$ $\uparrow$	S-measure$\uparrow$
$\checkmark$				0.034	**0.912**	0.843	0.881	0.904	0.051	**0.866**	0.78	0.813	**0.865**	0.031	0.947	0.898	0.93	0.928
$\checkmark$	$\checkmark$			0.033	0.908	0.849	0.88	0.905	0.051	0.865	0.788	0.814	**0.865**	0.029	0.947	0.904	0.930	0.929
$\checkmark$	$\checkmark$	$\checkmark$		0.033	0.908	0.849	0.88	0.904	0.051	0.863	0.787	0.812	0.864	0.029	0.947	0.905	0.93	0.929
$\checkmark$	$\checkmark$	$\checkmark$	$\checkmark$	**0.032**	**0.912**	**0.855**	**0.883**	**0.906**	**0.050**	0.865	**0.790**	**0.815**	0.864	0.028	**0.948**	0.907	**0.931**	**0.930**
(Ours:SU^2^GE-Net)																		
U^2^-Net				0.053	0.862	0.794	0.812	0.853	0.059	0.829	0.753	0.768	0.832	0.036	0.929	0.887	0.903	0.903
P2T-vgg				0.041	0.892	0.840	0.856	0.882	0.057	0.831	0.764	0.777	0.837	0.029	0.942	0.91	0.924	0.920
P2T-resnet				0.035	0.898	0.858	0.872	0.892	0.049	0.839	0.784	0.795	0.849	0.027	0.943	0.916	0.929	0.923
MSIN				0.037	0.884	0.828	0.825	0.884	0.055	0.810	0.756	0.738	0.833	0.028	0.935	0.908	0.899	0.920
SCRN				0.040	0.888	0.809	0.803	0.885	0.056	0.811	0.746	0.72	0.056	0.033	0.935	0.897	0.878	0.917
EGNet-R				0.039	0.889	0.815	0.816	0.887	0.053	0.815	0.756	0.738	0.053	0.031	0.935	0.901	0.887	0.918
BASNet				0.048	0.859	0.791	0.803	0.866	0.056	0.805	0.756	0.751	0.056	0.033	0.93	0.898	0.890	0.908
RCSB				0.035	0.889	0.840		0.881	0.049	0.809	0.752		0.835	**0.027**	0.938	**0.909**		0.919
DC-Net				0.035	0.899	0.852		0.896	0.053	0.827	0.772		0.849	**0.027**	0.942	**0.909**		0.924

Model				ECSSD					SOD					PASCAL-S				
Swin	TTA	Edge	GCT	MAE$\downarrow$	MaxF$\uparrow$	MeanF$\uparrow$	$F_{\beta }$ $\uparrow$	S-measure$\uparrow$	MAE$\downarrow$	MaxF$\uparrow$	MeanF$\uparrow$	$F_{\beta }$ $\uparrow$	S-measure$\uparrow$	MAE$\downarrow$	MaxF$\uparrow$	MeanF$\uparrow$	$F_{\beta }$ $\uparrow$	S-measure$\uparrow$
$\checkmark$				0.032	0.958	0.917	0.942	0.937	0.086	**0.883**	0.82	0.852	**0.830**	0.058	0.902	0.840	0.864	0.881
$\checkmark$	$\checkmark$			0.030	0.957	0.921	0.942	0.937	**0.083**	**0.883**	0.828	0.851	0.828	0.057	0.901	0.844	**0.865**	**0.881**
$\checkmark$	$\checkmark$	$\checkmark$		0.030	0.957	0.921	0.943	0.937	**0.083**	0.879	0.829	0.852	0.827	0.057	0.901	0.844	**0.865**	0.880
$\checkmark$	$\checkmark$	$\checkmark$	$\checkmark$	**0.028**	**0.959**	**0.925**	**0.945**	**0.939**	**0.083**	0.882	**0.834**	**0.854**	0.827	**0.055**	0.901	**0.847**	**0.865**	**0.881**
(Ours:SU^2^GE-Net)																		
U^2^-Net				0.041	0.947	0.907	0.922	0.915	0.119	0.859	0.772	0.785	0.770	0.084	0.865	0.797	0.808	0.829
P2T-vgg				0.034	0.956	0.925	0.938	0.928	0.102	0.871	0.808	0.818	0.797	0.065	0.888	0.837	0.848	0.860
P2T-resnet				0.032	0.953	0.927	0.938	0.927	0.098	0.871	0.818	0.828	0.799	0.062	0.887	0.845	0.855	0.864
MSIN				0.033	0.947	0.924	0.911	0.925						0.064	0.882	0.842	0.821	0.857
SCRN				0.037	0.950	0.918	0.899	0.927						0.065	0.890	0.839	0.816	0.867
EGNet-R				0.037	0.947	0.920	0.903	0.925						0.075	0.878	0.831	0.807	0.853
BASNet				0.037	0.942	0.879	0.904	0.916						0.077	0.863	0.781	0.800	0.837
RCSB				0.034	0.944	0.916		0.922						0.059	0.875	0.826		0.860
DC-Net				0.034	0.949	0.913		0.924						0.066	0.874	0.814		0.857

Higher values of *MaxF*, *MeanF*, $F_{\beta }$, and $S-measure$, and lower values of *MAE*, indicate better performance.

Optimal outcomes are highlighted in bold.

## Conclusions

Edge-based loss functions were designed to be trained for edges and use the GCT to promote cooperative and competitive relationships between neurons. Feature extraction is performed using the Swin TransformerV2. The non-specific class foreground subject segmentation algorithm SU$^2$GE-Net was proposed based on U$^2$-Net, and TTA loss was used to make the network converge more efficiently, solving the problems of concern Fig. 1 attention and edge roughness. The feasibility of edge-based loss computation was verified by showing edge detection results using the traditional edge operator for true label masks. Four different edge detection operators were also used for experiments, and the Canny operator with the best results was finally selected as the computation of the Edge Loss function. Validation using the multiple datasets demonstrated the excellent performance of SU$^2$GE-Net, which was better compared to some SOTA methods. The comparative experiments show that SU$^2$GE-Net has fast convergence and universal applicability when segmenting multiple image scenes. However, the model does not perform well in segmenting fine details such as hair, indicating a limitation in the size and type of the edge operator used in calculating the Edge Loss. We believe that future research should focus on proposing improved approaches to address this issue.

## Data Availability

The data generated and analysed during the current study are available from the corresponding author on reasonable request. The DUTS-TE datasets is available online at http://saliencydetection.net/duts. The ECSSD datasets is available online at https://datasets.activeloop.ai/docs/ml/datasets/ecssd-dataset/. The DUT-OMRON datasets is available online at http://saliencydetection.net/dut-omron/. The SOD datasets is available online at https://www.elderlab.yorku.ca/resources/salient-objects-dataset-sod/. The PASCAL-S datasets is available online at https://gas.graviti.com/dataset/graviti/PASCAL_S The HKU-IS datasets is available online at https://i.cs.hku.hk/yzyu/research/deep_saliency.html.
